# The Primary Transcriptome of *Salmonella enterica* Serovar Typhimurium and Its Dependence on ppGpp during Late Stationary Phase

**DOI:** 10.1371/journal.pone.0092690

**Published:** 2014-03-24

**Authors:** Vinoy K. Ramachandran, Neil Shearer, Arthur Thompson

**Affiliations:** 1 Department of Plant Sciences, University of Oxford, Oxford, United Kingdom; 2 Institute of Food Research, Norwich, United Kingdom; Animal Health and Veterinary Laboratories Agency, United Kingdom

## Abstract

We have used differential RNA-seq (dRNA-seq) to characterise the transcriptomic architecture of *S*. Typhimurium SL1344, and its dependence on the bacterial alarmone, guanosine tetraphosphate (ppGpp) during late stationary phase, (LSP). Under LSP conditions we were able to identify the transcriptional start sites (TSSs) for 53% of the *S*. Typhimurium open reading frames (ORFs) and discovered 282 candidate non-coding RNAs (ncRNAs). The mapping of LSP TSSs enabled a detailed comparison with a previous dRNA-seq study of the early stationary phase (ESP) transcriptional architecture of *S*. Typhimurium SL1344 and its dependence on ppGpp. For the purposes of this study, LSP was defined as an aerobic LB culture grown to a later optical density reading (OD_600_ = 3.6) compared to ESP (OD_600_ = 2.3). The precise nucleotide positions of the majority of *S*. Typhimurium TSSs at LSP agreed closely with those identified at ESP. However, the identification of TSSs at different positions, or where additional or fewer TSSs were found at LSP compared to ESP enabled the genome-wide categorisation of growth phase dependent changes in promoter structure, the first time such an analysis has been done on this scale. Comparison of the ppGpp-dependency LSP and ESP TSSs for mRNAs and ncRNAs revealed a similar breadth of ppGpp-activation and repression. However, we note several ncRNAs previously shown to be involved in virulence were highly ppGpp-dependent at LSP. Finally, although SPI1 was expressed at ESP, we found SPI1 was not as highly expressed at LSP, instead we observed elevated expression of SPI2 encoded genes. We therefore also report an analysis of SPI2 transcriptional architecture at LSP resulting in localisation of SsrB binding sites and identification of a previously unreported SPI2 TSS. We also show that ppGpp is required for nearly all of SPI2 expression at LSP as well as for expression of SPI1 at ESP.

## Background

Infections caused by non-typhoidal *Salmonella* are one of the most frequent causes of food-borne illness worldwide. Although it is difficult to estimate precisely due to unreported incidents, a recent report suggests that there are 93.8 million cases of gastroenteritis due to *Salmonella* species globally each year with 155,000 deaths [1].

In the present study we focus on *S*. Typhimurium, which mainly causes a self-limiting enterocolitis but can cause a more serious disease in immunosuppressed patients [2], [3]. Once ingested *S*. Typhimurium is able to penetrate intestinal epithelium cells, a process that is dependent on the expression of a type 3 secretion system (T3SS) encoded by a horizontally acquired set of virulence genes encoded within *Salmonella* Pathogenicity Island 1 (SPI1). In the case of immunocompromised humans, *S*. Typhimurium can become systemic resulting in a typhoid-like fever due to its ability to replicate and survive within macrophages; this is facilitated by the intracellular expression of a second T3SS encoded within SPI2 [4]. In the present study we focus on defining the transcriptomic architecture of *S*. Typhimurium during stationary phase. Stationary phase occurs when nutrients become scarce and bacteria develop a multiple-stress resistant state. Morphological changes are observed, including rounded shape, loss of flagella and thickening of the cell wall. General metabolism is redirected, macromolecular degradation is increased, and storage and osmoprotection compounds are synthesized [5]. The reorganization of the nucleoid is accompanied by an overall reprogramming of gene expression, much of it mediated by the bacterial alarmone ppGpp and the stationary phase sigma factor RpoS [6], [7]. The adaptation to stationary phase starvation and stress also involves the expression of virulence factors including genes encoded within SPI1 and SPI2, several of which have previously been shown to be ppGpp-dependently activated [23], [24], [26].

Guanosine tetraphosphate is produced by the RelA and SpoT enzymes in all beta- and gammaproteobacteria and whereas RelA only has ppGpp synthetic function, SpoT is able to both synthesise and hydrolyse ppGpp (for reviews see [7]–[9]). RelA mediates the stringent response during amino acid starvation and SpoT modulates ppGpp levels in response to a variety of stresses including phosphorus, nitrogen carbon, iron, fatty acid limitation. The effect of ppGpp occurs via direct binding to RNA polymerase (RNAP) resulting in the modulation of transcription [10]. Interestingly, it has also been shown that ppGpp plays a key role in coupling virulence to metabolic status in several pathogenic bacteria including *Mycobacterium tuberculosis* [11], [12], *Listeria monocytogenes* [13], *Legionella pneumophilia* [14], [15], *Vibrio cholera* [16] and *Pseudomonas aeruginosa* [17]. In *S*. Typhimurium, virulence in Balb/C mice was shown to be completely dependent on ppGpp due to the presence of SpoT rather than RelA [18]. The transcriptomic architecture of *S*. Typhimurium at ESP during expression SPI1 and its dependence on ppGpp, has previously been defined using dRNA-seq resulting in the definition of TSSs for the majority of *S*. Typhimurium genes and the discovery of many new ncRNAs [19], [20]. In the present study we used dRNA-seq to define the transcriptomic architecture of *S*. Typhimurium and its dependence on ppGpp during later stationary phase when the expression of SPI2 encoded genes are elevated, in addition to changes in the expression patterns of many other genes and ncRNAs involved in the transition to a nutrient deprived and microaerophilic environment.

Our previous analysis of the transcriptomic architecture of *S*. Typhimurium during early stationary phase (ESP) identified the TSSs for 78% of the *S*. Typhimurium genome [19]. In the current study we define the TSSs for 53% of the *S*. Typhimurium genome during LSP and show that the majority of LSP TSS positions agree to within 0–3 nt of our previously defined ESP TSSs. However, we found that 13.5% of the LSP TSSs were present at LSP and not ESP or had differently positioned TSSs compared to previously defined ESP TSSs [19]. We report the discovery of 282 new candidate ncRNAs of which 159 were antisense RNAs (asRNAs); five of the asRNAs were located opposite SPI2 encoded genes. Finally, the transcriptomic architecture of *S*. Typhimurium at stationary phase was found to be highly ppGpp-dependent with 33% of the TSSs for protein coding genes and 29.4% of the candidate LSP ncRNAs being directly or indirectly controlled by ppGpp.

## Results

### Identification of stationary phase transcriptional start site positions and operons

A dRNA-seq approach was used to identify the precise nucleotide positions of TSSs from RNA samples isolated from an *S*. Typhimurium parental strain, SL1344, and an isogenic Δ*relA*Δ*spoT* strain grown aerobically to LSP in LB. The respective growth curves for the parental and Δ*relA*Δ*spoT* strains were shown to be almost identical (Fig. S1). The growth point (OD_600_ = 3.6) and time at which samples were taken for dRNA-seq analysis are indicated in Fig. S1. A description of how dRNA-seq (differential RNA-seq) was used to define TSSs position is described in [19], [21]. Briefly, the procedure involves preparing two cDNA libraries from the same RNA sample, one of which is enriched for primary transcripts by treating with terminator exonuclease which specifically degrades processed transcripts. Comparison with the untreated library reveals the location of TSSs due to the elevated read numbers of transcripts from this library compared to the treated library. The mapped reads from this study and also from the ESP dataset [19] can be viewed on JBrowse (http://jbrowse.org/) by following the link provided at www.ifr.ac.uk/Safety/MolMicro/.

**Figure 1:**
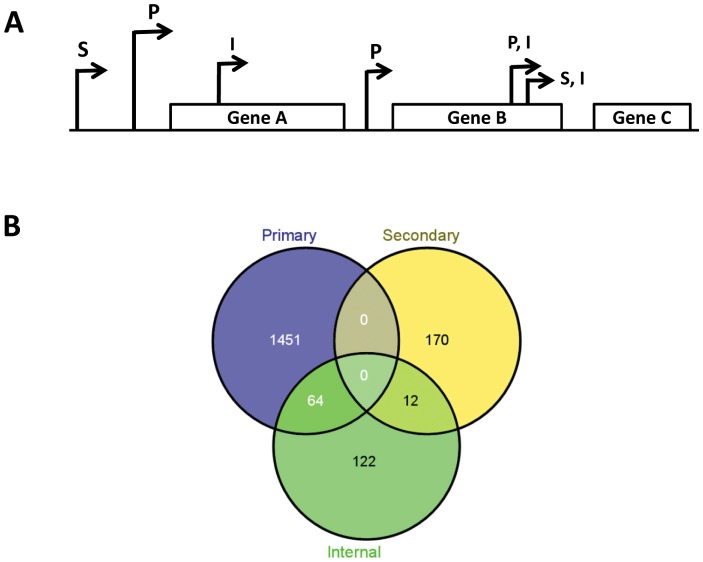
Annotation of TSSs. (A) TSSs were defined as primary (P), secondary (S) or internal (I). Primary TSSs were identified as having higher mapped read counts relative to secondary TSSs. Internal TSSs were located within the coding region (CDS) of a gene where a TSS was annotated for the gene immediately upstream. Primary and secondary internal TSSs (P,I and S,I respectively) were located within the CDS of a gene where there were no TSSs annotated for the gene immediately downstream. (B) Venn diagram showing overlap of *S*. Typhimurium LSP TSSs categories for all annotated genes.

We mapped a total of 2186 LSP TSS's on to the SL1344 chromosome (including all annotated ORF's, stable RNAs and ncRNAs and a total of 78 for the SLP1–3 endogenous plasmids (Tables S1, S2, S3, S4, S8). The TSSs were categorised as primary, secondary, internal or present only in the Δ*relA*Δ*spoT* strain; however many had multiple associations and these are defined and summarised in Fig. 1. Primary LSP TSSs were identified for a total of 2538 mRNAs (including intra-operonic genes) representing 53% of the annotated SL1344 genome (Genebank ID FQ312003.1; Table S5). The proportion of genes for which LSP TSSs were identified was lower than was previously defined for ESP (78%) [19], most likely due to restricted growth and metabolic activities at LSP; however a comparison of the TSS positions for mRNAs found at ESP and LSP revealed that 86.5% (1576) of LSP TSSs were either identical or within 1 to 3 nt of the previously defined TSS positions for ESP (Table S1) [19]. Of the remaining 13.5% of the LSP TSSs, 6.0% (111) were specific to LSP (i.e. found within genes for which no ESP TSSs had previously been defined), and 7.5% (134) were repositioned by 10 nt or greater (median = 63 nt) at LSP compared to ESP (Table S2) [19]. The accuracy of dRNA-seq for the identification of TSSs (92% of ESP TSSs were located within 10 nt of experimentally determined TSSs [19]) suggests that the repositioning of 7.5% of the LSP TSSs relative to their ESP TSS positions was not due to experimental inaccuracies. A large number (924) of the chromosomal TSSs and 23 SLP TSSs that had previously been found at ESP were not present at LSP, nor was there any alternative LSP TSS, suggesting the corresponding genes were not transcribed at LSP or were subject to other regulatory mechanisms (Table S7). A previous DOOR-based prediction of operon structure inferred from ESP dRNA-seq data revealed 625 predicted operons [19]. Here we confirmed the structure of the predicted ESP operons and in addition defined 46 operon structures specific to LSP (Table S1). Finally, it has long been known that the majority of TSSs start with a purine residue, and analysis of the TSS located upstream of annotated LSP ORF's (Table S1) revealed that 71% of the transcripts started with a purine residue (A - 46%, G – 25%), in agreement with the known preference for a purine residue at the +1 position [22] (data not shown).

### Transcriptomic architecture of SPI2 during late stationary phase

**Figure 2:**
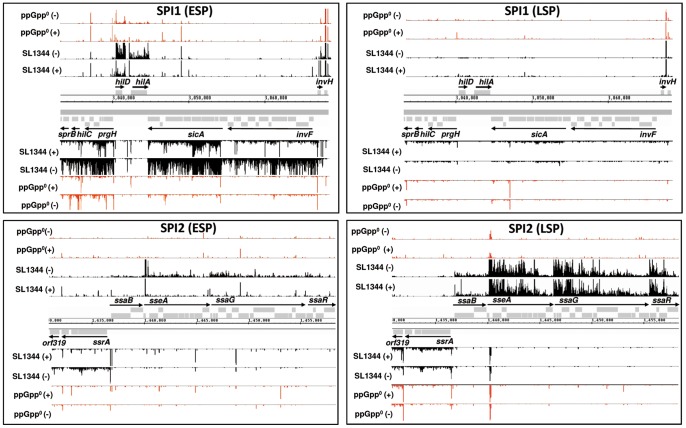
Transcriptional architecture of the entire SPI1 and SPI2 loci at ESP and LSP in parental and Δ*relA*Δ*spoT* strains. Enriched (+) and non-enriched (−) cDNAs of *S*. Typhimurium parental (black) or ppGpp^0^ (*ΔrelAΔspoT*) (red) strains mapped onto the SPI1 and SPI2 loci. Operons are indicated by horizontal arrows and annotated according to the first gene. The Y axis in each lane represents 0–50 mapped reads per genome position. The dRNA-seq data for ESP was from [19].

SPI2 is expressed during intracellular replication of *S*. Typhimurium within host cells [4]. Whist stationary phase culture in LB is unlikely to represent a physiologically apt model of the intracellular environment, we nevertheless observed that the expression of the majority of SPI2 but not SPI1 genes were elevated under LSP conditions compared to ESP; this expression pattern has previously been noted and was also verified from a microarray based transcriptomic analysis shown in Fig. S6 [19], [20], [23]–[26]. The consecutive expression pattern of SPI1 followed by SPI2 expression during transit through stationary phase (Fig. 2 and Fig. S6) mimics the infection situation whereby SPI1 largely precedes SPI2 expression and they rarely occur simultaneously in the same niche [27]. The elevated expression of SPI1 (but not SPI2) at ESP was defined by the increased reads mapping to the SPI1 locus (Fig. 2AC; data from [19]). Conversely, elevated SPI2 expression at LSP (but not SPI1) is shown in Fig. 2CD, confirming previous findings [23]–[26], and verified in Fig. S6. During both ESP and LSP, Fig. 2 and Fig. S6 also clearly show that SPI1 and SPI2 expression were dependent on ppGpp for activation. The expression of SPI2 during LSP enabled us to undertake an analysis of the transcriptomic architecture of SPI2 under these conditions.

**Figure 3:**
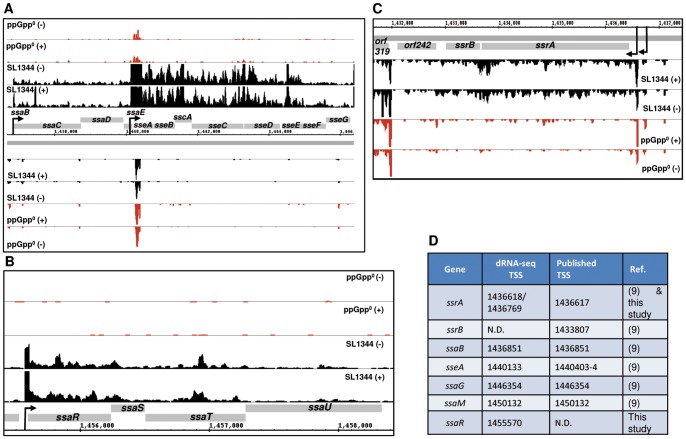
Promoter architecture and ppGpp-dependency of SPI2. Enriched (+) and non-enriched (−) cDNAs of *S*. Typhimurium SL1344 parent (black) or ppGpp^0^ (*ΔrelAΔspoT*) (red) strains mapped onto selected regions of the SPI2 locus. Operons are indicated by horizontal arrows and annotated according to the first gene. Transcript start sites are indicated by black arrows (A) Map of *ssaB* and *sseA* operons. The Y axis in each lane represents 0–50 mapped reads per genome position. (B) Map of *ssaR* operon. The Y axis in each lane represents 0–150 reads. (C) Map of the *ssrA* operon showing transcriptional read through into *ssrB* and *orf242*. Y axis in each lane represents 0–30 reads. (D) Location of transcript start sites identified previously and in this study; N.D.  =  not determined.

Due to its importance in *Salmonella* pathogenicity, SPI2 has been widely studied in terms of its organisation and regulation and in the role of individual effector proteins. However we were able to gain new insights into features of SPI2 regulation from a comparison of our dRNA-seq data under SPI2 inducing conditions with previously published work. Analysis of the dRNA-seq data confirmed a TSS 167 nt upstream of the *ssrA* translational start at genomic position 1436618 (only 1 nt distant from a previously reported *ssrA* TSS [28], and in addition, confirmed the position of a second previously reported TSS at genomic position 1436769 (Fig. 3CD, [19]). No TSSs could be identified immediately upstream of *ssrB* or *orf242* (SL1324), although sequencing reads extended into both ORFs implying that under the growth conditions used here, they were co-transcribed with *ssrA* (Fig. 3C). Further examination of the dRNA-seq data identified TSSs upstream of *ssaB*, *ssaG*, *ssaM* and *ssaR* (Fig. 3ABD, Table S1). The TSSs for *ssaB*, *ssaG* and *ssaM* agree closely with those determined experimentally (Fig. 3D) [28], [29]. The *ssaR* TSS has not previously been identified although promoter activity and SsrB binding in the *ssaR* upstream region has been reported and a consensus SsrB binding site was identified upstream of the *ssaR* TSS [30] (Table S6).

The *sseA* TSS has previously been mapped at two adjacent sites at genomic positions 1440403-4, [29], (Fig. 3D). We were unable to clearly identify a TSS for *sseA* at this position in the LSP dRNA-seq data, however, according to data from ESP, an additional TSS for *sseA* was identified within the 3′ end of the *ssaE* coding region at genomic position 1440133, which lies immediately upstream of *sseA* [19], (Fig. 3AD). We also note an SsrB binding site 36 nt from this TSS, shown in Table S6. In the LSP dRNA-seq data a sudden increase in transcript levels was observed within a few nt's of the TSS internal to *ssaE* (Fig. 3AD), making it highly likely that this is the TSS for *sseA*; however, the precise position of the TSS may be obscured in the LSP sequencing data, possibly due to interaction with other regulatory mechanisms. Alternately, or in addition, the transcript levels of the *ssaE* operon may be being affected by a ∼150 nt transcript which was observed anti-sense to the *ssaE*-*sseA* intergenic region (Fig. 3A); we are currently investigating this possibility.

The SPI2 operons are all activated by SsrB and yet many SPI2 encoded genes are also controlled either directly or indirectly by multiple ancestral proteins [31]. Knowledge of the precise location at which SsrB binds to its dependent promoters in relation to RNA polymerase will facilitate research on the interaction between the different regulatory components. Where they have been mapped, SsrB dependent promoters have been found to be diversely organised with SsrB binding to sites upstream, overlapping or downstream of the TSSs of different genes [29]. Although for some genes it has been noted that SsrB can directly activate transcription by binding immediately upstream of their promoters [29], a full analysis of SsrB dependent promoters has been limited by the number of targets for which both SsrB binding site and TSS information was available. The dRNA-seq analysis presented here and published previously [19] has extended the number of SsrB dependent promoters for which TSSs have been mapped. We combined this information with previously published TSSs and SsrB binding sites [30] to map the promoters of all the SPI2 operons and 14 additional SsrB regulated operons encoded outside of the SPI2 locus (Table S6). This analysis revealed that the majority of SsrB binding sites were located immediately upstream to, or overlapping a region 35 nt upstream of the TSS (Table S6). At promoters where SsrB does not appear to bind close to the −35 region it is also possible that SsrB activates transcription to initiate at alternative TSSs used only under certain growth conditions; for example, we identified a weak TSS signal 34 nt downstream of a consensus SsrB binding site at the *sseI* promoter, although the previously reported TSS is located 100 nt upstream of the SsrB binding site (Table S6).

### Comparison of ESP and LSP TSS positions

**Figure 4:**
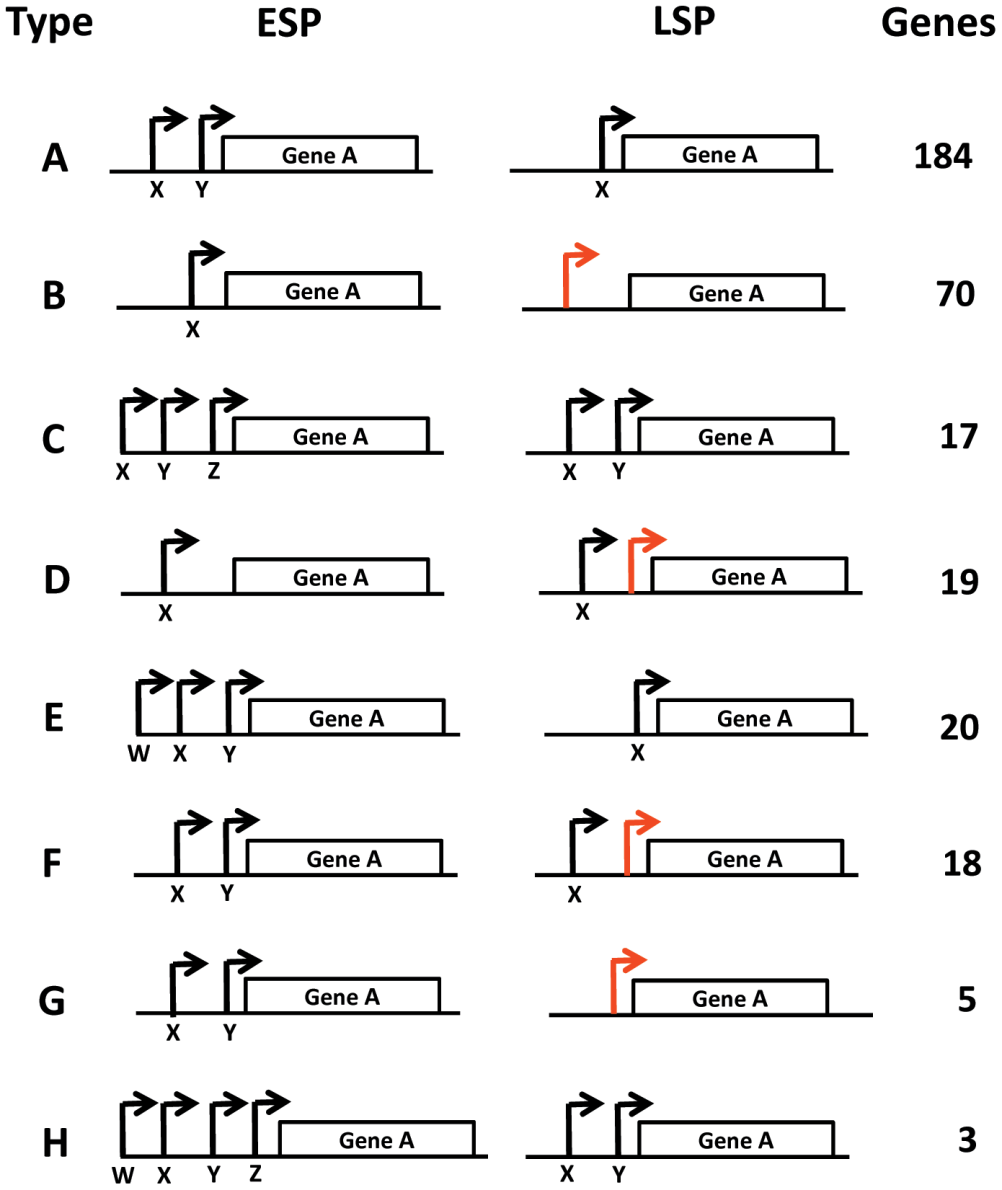
Use of alternate TSSs at LSP compared to ESP. Letters A to H annotate the different categories of arrangements of TSSs found at LSP compared to ESP that we were able to identify. Letters X, Y, Z and W represent TSS positions at ESP and LSP. The number of genes for which each type of rearrangement occurs is shown. For nucleotide positions of TSSs and genes to which the different types of rearrangements apply see Table S2. Red arrows indicate TSS positions that were identified at LSP but not ESP. Rearrangements which apply to one or two genes are shown in Fig. S2.

The LSP mRNA TSSs were compared to our previously identified ESP TSSs ([19], Tables S1, S2). Although a close correlation was found between the majority of ESP and LSP TSS positions, we found 134 TSSs which had different positions at LSP compared to ESP (>10 nt); these will be discussed in the next section of the results. In addition we found 111 LSP-specific TSSs for which no previous ESP TSSs had been identified, [19], Fig. 4, Fig. S2 and Table S2). The 111 LSP-specific TSSs were found within the promoters for 105 genes. A survey of the functions of the 105 genes revealed that several were anaerobically induced and involved in fermentative metabolism (*adhC*, *citA*, *fucR*, *napB, fdoG, pflD*), as might be expected to occur in a stationary phase microaerophilic environment. At LSP, lack of nutrients is also a limiting growth factor and this was suggested by the presence of LSP TSSs for genes encoding gluconate, glucose and iron transporters (*glnR*, *idnT*, *ptsG, entC, sitA*). A low phosphate and magnesium environment is suggested by the presence of LSP specific TSSs for *pstB*, which encodes a phosphate transport ATP-binding protein [32], and *rstB* which is the sensor kinase in a two component system (with RstA) [33]. Expression of *rstB* is induced under low magnesium growth conditions through the PhoPQ two-component system [34]. The RstA/B regulon is induced at stationary phase and includes genes involved in acid tolerance, curli fimbria formation and anaerobic respiration [33]. RstAB and PhoPQ are thought to form a signal relay cascade involved in the adaptive response to acid conditions [35]. RstA expression is also able to promote RpoS degradation in *S*. Typhimurium and has been shown to be involved in modulating *Salmonella* biofilm formation [36]. The low pH, phosphate and magnesium environment implied at LSP by the identification of TSSs for *pstB* and *rstB* has also been shown to trigger activation of SPI2 under minimal media growth conditions [25], [37]–[39].

### Promoters with differing TSSs at LSP and ESP

A comparison of the TSSs at LSP and ESP found within the same promoters was performed and revealed that 134 TSSs either differed in position (>10 nt) and/or were found to have fewer or more TSSs at LSP compared to ESP (Fig. 4). For ease of reference the different types of rearrangements of TSSs between LSP and ESP, along with the numbers of genes associated with each type of rearrangement were categorised alphabetically (Fig. 4; Fig. S2 also shows rearrangements that occurred in one or two genes only). The genomic positions of the TSSs at LSP, relative to ESP and their corresponding genes and function, are shown in Table S2. In summary, we found the majority of differences between ESP and LSP TSS positions occurred where one or more of the ESP TSSs were not present at LSP (274 TSSs) and these constituted types A, C, E and H shown in Fig. 4 (and M, N in Fig. S2). We identified 84 LSP TSSs where the ESP-defined TSS was absent but was instead replaced by a differently positioned TSS at LSP (shown by red arrows in Fig. 4); these constituted types B and G in Fig. 4 (and J, K, L in Fig. S2). We also identified 37 genes which had LSP TSSs in addition to one or more ESP TSSs; these constituted types D and F and are also shown by red arrows in Fig. 4. Finally we found that in the case of 17 genes, TSS position was ppGpp-dependent (categorised as ‘R’ in Fig. S2 and Table S2).

Interestingly several genes in which one or more ESP TSSs were absent at LSP (and therefore presumably not used at LSP) had virulence related functions; these included the SPI1 regulators *hilC* and *hilD* and also the SPI1 encoded genes *prgH,* and *invH* (categorised as type ‘A’ in Fig. 4 and Table S2). Other virulence related regulators in which one or more TSSs were absent at LSP compared to ESP included *csrA, dksA, hfq*, *hns, mig-14, pagK, pipA* (SPI5), *slyA and virK*. Similarly we could also identify 70 genes at LSP where the ESP TSS was absent but where an LSP TSS at a differing position was present (designated as type ‘B’ in Fig. 4 and Table S2). A survey of the functions of the latter genes revealed several with metabolic, virulence and regulatory functions. These included the anti-sigma factor encoding genes *rseE* and *rseA*, which are involved in sigma E activation in response to envelope and acid stress, and involved in virulence in *S*. Typhimurium [40], [41]. Other virulence related genes of type B where the ESP TSS was absent and the LSP TSS was in a different position included *lrp, pipD* (SPI5), *pagC, ygdP* and *rfaH.* Lrp has been shown to affect many stationary phase induced genes including those involved in the response to nutrient limitation, high concentrations of organic acids, and osmotic stress [42]. Lrp has also been shown to act as a virulence repressor in *S*. Typhimurium [43]. YgdP is a dinucleoside polyphosphate hydrolase, mutant alleles of *ygdP* have been shown to reduce invasion of *Salmonella* by repressing SPI1 expression [44]. RfaH encodes a stationary phase-expressed transcriptional anti-terminator and affects operons encoding extracytoplasmic cell components involved in the virulence of *E. coli* pathogens and has also been shown to down-regulate key virulence factors in *S*. Typhimurium [45], [46].

### Non-coding RNAs at stationary phase

The new candidate LSP ncRNAs are listed in Table S3. Candidate ncRNAs were defined as either intergenic (>250 nt from a TSS) or antisense (strictly defined as being opposite the CDS of a gene). We found a total of 282 candidate ncRNAs of which 159 were asRNAs and 123 which were located in intergenic regions. Candidate asRNAs were found to 5 SPI2 genes, 4 of which encode secretion system effectors (*ssaB*, *ssaC*, *ssaV* and *sseG*), and *orf32*. Other virulence related genes with candidate asRNAs included *virK*, *stcD*, *mgtB* and two genes related to iron transport: *iroC* and *fhuE* (Table S3). A MEME analysis of the 282 candidate ncRNAs revealed conservation of a consensus sequence closely matching the *E. coli* σ^70^ (−10) binding site (TATAAT), (Fig. S3). An analysis of the first nucleotide of the candidate LSP ncRNA transcripts also revealed a tendency for a purine residue (A – 41%, G – 29%), as has been previously noted for the +1 position of TSSs [22] (data not shown). Finally, we determined the LSP read counts for the 114 sRNAs which have previously been validated by Northern blot analysis (Table S8, [20]). We were able to identify TSSs for all but 21 of the validated sRNAs (for read counts >3). Several of the validated sRNAs with the highest read numbers at LSP (SdsR, CsrB, CsrC, RprA, GlmY and MgrR) have been shown to be highly expressed during stationary phase; CsrB and CsrC have both been shown to play a role in virulence in *Salmonella* and MgrR shown to play a role in sensitivity to antimicrobial peptides in *E. coli* [47]–[49].

### Defining ppGpp-dependent gene expression at stationary phase using dRNA-seq

**Figure 5:**
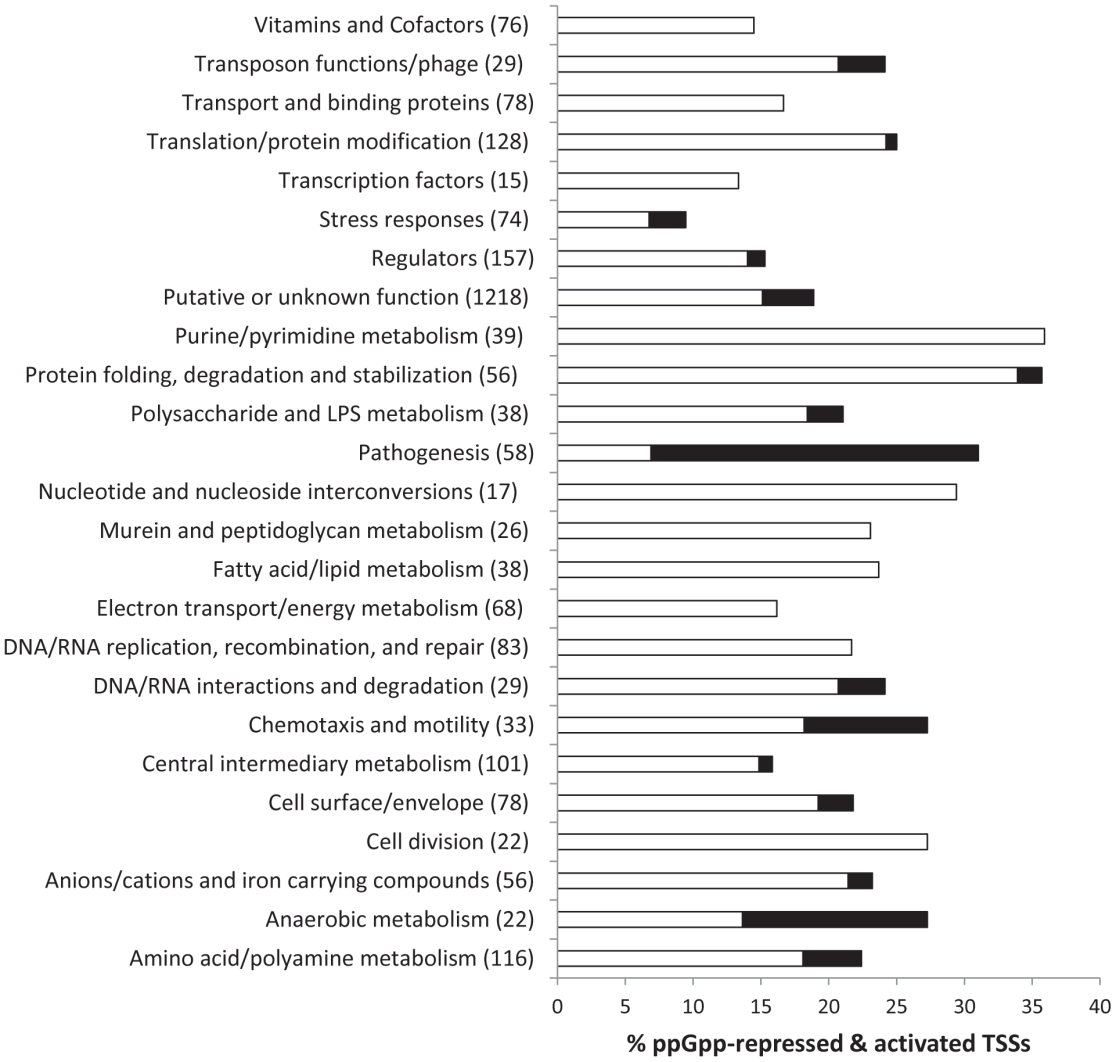
Functional category analysis of ppGpp-dependent genes. Functional categories were compiled from the Kyoto Encyclopedia of Genes and Genomes (KEGG; www.genome.jp/kegg) and The Comprehensive Microbial Resource (CMR) at the J. Craig Ventner Institute (http://cmr.jcvi.org/tigr-scripts/CMR/CmrHomePage.cgi) and a manual inspection based on the published literature. The total number of ORFs present in each category is indicated in parentheses.

The dRNA-seq analysis revealed that the positions of 17 mRNA TSSs were ppGpp-dependent (categorised as type ‘R’ in Fig. S2 and listed in Table S2); an example of a ppGpp-dependent alteration in TSS position is shown for *gltA* which encodes citrate synthase (Fig. S4). Whether the ppGpp-dependent alterations in TSS position have any regulatory significance remains to be determined. We also observed large ppGpp-dependent changes in the read counts mapping to the TSSs identified at LSP (Table S1). We interpreted this as ppGpp-dependent transcriptional regulation; RNA-seq data has been used previously to identify differences in the transcriptional status of bacterial genes and validated by comparison with microarray experiments [50]–[52]. In order to estimate the expression level of a promoter from dRNA-seq data we calculated the number of non-enriched reads mapping between the primary TSS and 50 nt downstream of the TSS. ppGpp-activated expression was defined as 4-fold or higher transcript levels in the parent strain compared to the Δ*relA*Δ*spoT* strain and ppGpp-repressed expression was defined as 4-fold or higher transcript levels in the Δ*relA*Δ*spoT* strain compared to the parent strain (Table 1). For LSP mRNAs, we identified a total of 511 TSSs that were ppGpp-repressed by 4-fold or greater; these included 88 TSSs for which no mapped reads could be discerned in the SL1344 parent compared to the Δ*relA*Δ*spoT* datasets. Only 96 (5.2%) TSSs were found to be ppGpp-activated by 4-fold or greater. In total 33% of the TSSs for protein coding genes were ppGpp-dependent at LSP compared to 34% at ESP; together these are indicative of the major role ppGpp plays in modulating the stationary phase gene expression programmes (Table 1 and Table S1). The genes for the ppGpp-repressed TSSs were categorised according to function (Fig. 5), and the majority (184 genes) were of putative or unknown function. The largest functional classes of ppGpp-repressed genes were involved in purine/pyrimidine metabolism (36%), protein folding, degradation and stabilisation (34%) and nucleotide and nucleoside interconversions (29%) (Fig. 5). Other highly represented classes of ppGpp-repressed genes were involved in translation and protein modification (24%) and fatty acid and lipid metabolism (24%). Of the 92 genes that were ppGpp-activated greater than 4-fold, the majority were of putative or unknown function however the next largest category (13 genes; 22%) had virulence functions and included 5 SPI2 genes (*ssaB, ssaG, sseA, sseI, sseJ, sseL*) (Table 1 and Table S1).

**Table 1:** The relative proportions of ppGpp-repressed and ppGpp-activated TSSs within the *S*. Typhimurium genome at LSP.

TSSs type	% ppGpp-repressed	% ppGpp activated	Total TSSs
ORF P	34	6	1453
ORF P, I	34	8	64
ORF S	34	1	170
ORF S, I	33	0	12
ORF I	16	8	122
asRNAs	28	6	159
Other ncRNAs	15	10	123
Validated ncRNAs	12	11	105
tRNAs	22	10	40
rRNAs	86	0	14

Compiled from Tables S1, S3, S4 & S8.

For the stable RNA genes, the TSSs for all but 2 of the operons containing rRNA genes were ppGpp-repressed (greater than 4-fold), consistent with the ppGpp-repression of TSSs for genes involved in translation/protein modification described above, and with the general down-regulation of protein synthesis occurring at LSP (Table S4). However, in contrast to the general trend of ppGpp-repression of stable RNA genes, we found 7 tRNA genes, cognate to leucine, arginine, and tyrosine which were ppGpp-activated greater than 4-fold (Table S4).

**Figure 6:**
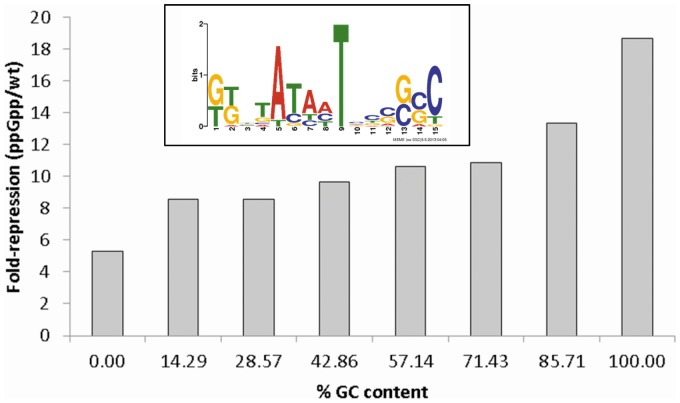
Correlation of ppGpp-repression of LSP TSSs of annotated genes with %GC content of discriminator regions. The analysis of GC content within the discriminator region was calculated using a custom Perl script. The inset shows the GC rich conserved region identified using MEME within the discriminator regions of promoters that were ppGpp-repressed by 20-fold or greater.

It is known that ppGpp-repressed promoters tend to contain GC rich discriminator regions between the TSS and −10 regions that play a role in destabilising the RNAP- promoter complex resulting in transcriptional repression (e.g. rRNA promoters [53], [54]). We therefore compared the GC content of the discriminator regions for all of the LSP TSSs to the level of repression by ppGpp. The median fold-repression of all of the ppGpp-repressed TSSs compared to GC content revealed a bias towards ppGpp-dependency with higher GC content (Fig. 6). A MEME comparison of the discriminator regions upstream of all of the ppGpp-repressed TSSs revealed no conserved region, however, 23 TSSs (out of 41) that were ppGpp repressed greater than 20-fold revealed a GC rich conserved region (Fig. 6, inset).

### ppGpp-dependent ncRNA's at stationary phase

Of the 282 LSP candidate ncRNAs 22% (63) were ppGpp-repressed by a factor of 4-fold or higher; this is more than double the total proportion of ppGpp-repressed ESP ncRNAs (10.2%, [19], Table 1 and Table S3). We also found 7.4% (21) of the LSP ncRNAs were ppGpp-activated by a factor of 4-fold or higher which was slightly lower than the proportion of ppGpp-activated ncRNAs at ESP (10.2%). (Table 1 and Table S3, [19]). In comparison to the ppGpp-dependent TSSs observed for LSP mRNAs, the total proportion of ppGpp-dependent TSSs for LSP candidate ncRNAs was similar (33% and 29.4% respectively for mRNAs and candidate ncRNAs respectively), however the overall range of ppGpp-dependency was smaller for ncRNA TSSs compared to mRNA TSSs. A MEME analysis of all of the ppGpp-repressed LSP ncRNAs failed to reveal a conserved GC rich region within the discriminator region however, this may be due to the small size of the dataset or indirect regulation by ppGpp. The sRNAs previously validated by Northern blot [20] displayed a similar pattern of ppGpp-dependency to the candidate LSP ncRNAs (Table S8). We found 11.4% (12) of the validated sRNAs were ppGpp-repressed and 10.5% (11) were ppGpp-activated (Table S8). The most highly ppGpp-repressed sRNAs (>4-fold) were CyaR, SraB, RydB, GcvB, SsrA, RyfA, OmrB and SsrS. Both CyaR and OmrB have been shown to down-regulate outer membrane porins [55]–[57], and SraB has been shown to affect survival of *S.* Enteritidis in response to antibiotics in egg albumin [58]. GcvB has been shown to be an important regulatory node in amino acid metabolism as well as limiting amino acid uptake [59], [60]. The validated ppGpp-induced sRNAs included InvR, RygD and RybB (Table S8). Interestingly InvR was the most highly ppGpp-induced sRNA at LSP (41-fold) and is encoded within SPI1, although has not been shown to play a role in the SPI1 secretion pathway or invasion. InvR and RybB have both been shown to repress the synthesis of the abundant outer membrane porin protein OmpD [61], [62].

## Discussion

The definition of the transcriptomic architecture for *S*. Typhimurium at later stationary phase resulted in the prediction of TSSs for 53% of ORFs which were found to closely correlate with the TSS positions for these ORFs previously defined at ESP [19]. The fewer mRNA TSSs that we were able to identify at LSP compared to ESP (53% and 78% respectively) are likely to be representative of the regulatory changes accompanying the decreased transcription occurring at LSP. However, we were able to define 245 LSP specific TSSs and found that 111 of these were within genes which had no identifiable TSS at ESP; the remaining 134 TSSs differed in position between LSP and ESP suggestive of the regulatory changes occurring between these growth phases. Functionally, the genes with TSSs that were either specific to LSP (i.e. not present at ESP), or in a different position compared to ESP TSSs were mostly involved in metabolic and regulatory changes which could be related to nutritional and other stresses encountered at stationary phase, and also within several virulence-related genes.

We were also able to map the transcriptomic architecture of SPI2 at LSP and identify a previously unreported TSS for *ssaR*. We were unable to identify a TSS upstream of *ssrB* or *orf242*, suggesting they were co-transcribed with *ssrA* (Fig. 3C). Feng and colleagues [28], found *ssrA* and *ssrB* expression to be uncoupled, with *ssrB* having its own promoter which was dependent on both OmpR and SsrB. However, it appears that this promoter is only active under specific growth conditions since other workers found no evidence of SsrB binding or functional promoter activity directly upstream of *ssrB* [30], [63]. The finding that *orf242* is apparently co-transcribed with *ssrAB* is potentially interesting since a previous study found no evidence of it being required for virulence [64]. The mapping of TSSs for SsrB-dependent genes enabled us to locate the relative positions for predicted SsrB binding sites within their promoters, revealing that most sites were located close to or overlapping the -35 regions suggesting that SsrB activates transcription through direct interactions with RNA polymerase using a CRP-like Class II activation mechanism (reviewed in [65]). Alternative architecture at other promoters suggests SsrB can employ additional activation mechanisms, for example at the *slrP* promoter where the SsrB binding site overlaps the TSS (Table S6). Based on a MEME and FIMO analysis (meme.nbcr.net/meme/fimo-intro.html), we were unable to identify any candidate SsrB binding sites or close matches within the promoters of the candidate and ‘validated’ ncRNAs described here and in [19], [20].

We report the discovery of 282 new candidate ncRNAs at LSP and 159 of these were asRNAs. The remaining candidate LSP ncRNAs (123) were located in the intergenic regions of genes and we note that 16 and 3 candidate ncRNAs were opposite the 5′ and 3′ regions of genes respectively and hence may play a *cis*-acting regulatory role in the expression of these genes. Indeed, several of the candidate ncRNAs were located opposite the 5′ regions or CDS's of genes involved in virulence or combating stress; for example we found two ncRNAs (LSPncRNA41and LSPncRNA39) which were opposite the 5′ UTRs of *ahpC* and *entC* repectively, the former encodes a subunit of alkyl hydroperoxide reductase involved in combating oxidative stress and the latter encodes the bacterial siderophore enterochelin, involved in iron uptake within the host [66]. Oxidative stress has been noted to be present at stationary phase [67]. We were able to identify TSSs for the majority of sRNAs previously validated by Northern blots and found that some of the highest read numbers mapped to sRNAs that have been shown to play a role in adaptation to stationary phase or in *S*. Typhimurium virulence (Table S8). SdsR sRNA was the most abundant later stationary phase sRNA and has been shown to down-regulate the major outer membrane porin, OmpD in *S*. Typhimurium [68]. We also note that CsrB and CsrC were amongst the most highly expressed sRNAs at LSP (Table S8). The CsrB and CsrC sRNAs antagonise CsrA which has many regulatory targets including biofilm formation, motility, virulence and metabolism, (for reviews see [69], [70]). Both CsrB and CsrC were found to be strongly induced during growth in nutrient-poor medium, and it has been suggested that together with the BarA-SirA system, may form a mechanism by which bacteria sense the energy/growth status of the cell and switch from growth on glycolytic to gluconeogenic substrates which occurs at stationary phase [71], [72]. Interestingly we found the TSS for CsrB but not CsrC to be ppGpp-repressed by a factor of 3.65-fold at LSP, (and 3.56-fold at ESP [19]), suggesting an indirect mechanism by which ppGpp could post-transcriptionally affect the function of many cellular processes in response to changes in the amino acid availability [71]. Several sRNAs have been shown to be encoded within *Salmonella* pathogenicity islands [73] and we were able to identify TSSs to four of these (IsrB-1, IsrJ, IsrK and IsrH-2; Table S8). IsrJ has been implicated in the invasion of *Salmonella* into nonphagocytic cells and IsrH was found to be up-regulated during infection of J774 macrophages; IsrB was upregulated when cells were grown in minimal media and IsrH was up-regulated under a variety of stress conditions and during stationary phase [73]. Finally, we report two LSP ncRNAs that were located on the opposite strand to other ncRNAs: LSPncRNA125 and LSPncRNA312 were opposite the previously validated sRNAs Stnc710 and Stnc1060 respectively [19], [20]. Several other examples of opposing ncRNAs have previously been noted potentially adding to the complexity of post-transcriptional regulatory mechanisms [19].

An analysis of the ppGpp-dependence of LSP mRNA TSSs showed very similar levels of ppGpp-repression compared to ESP TSSs (28% and 27% respectively, [19]). This is likely to be indicative of the major role ppGpp plays in modulating stationary phase gene expression, however not all of the ppGpp-repressed TSSs found at ESP were repressed at LSP and *vice versa*. These observations support evidence suggesting that although ppGpp plays a major role in transcriptional regulation, its action is essentially passive and modified by other regulatory elements such as sigma factors [74]. Fig. 2 reveals the reduced read numbers mapping to SPI1 TSSs at LSP compared to SPI1 at ESP [19]. At LSP there were no identifiable TSSs for *hilA* or *rtsA* and neither could we identify a TSS for *hilD* in the parental strain at LSP (Table S7). RtsA is a transcriptional activator of *hilA* and HilA is a major activator of SPI1 genes; HilD is the coordination point for several regulators that operate at the translational and transcriptional levels to control SPI1 and SPI2 expression [75]. Interestingly we observed a TSS with a perfectly conserved -10 region (TATAAT) in the Δ*relA*Δ*spoT* strain at genomic coordinate 3039861 which was 552 nt upstream of the translational start site of *hilD* and opposite to the CDS of *prgH* (Fig. S5).This TSS may represent a potential ppGpp-dependent start site for *hilD*, or spurious transcription, or promote an antisense transcript to *prgH* (TSS position 304128). We note that this TSS was also identified as ppGpp-repressed at ESP with very similar read numbers compared to LSP [19]. In addition we also identified a TSS mapping to genomic coordinate 3047036 within SPI1 at LSP which had higher read counts in the Δ*relA*Δ*spoT* strain compared to the parent strain (Fig. S5). The latter TSS was internal to the *sipF* coding region and upstream of *sicP* and *sptP*. We also note that this TSS was also identified at ESP, but was ppGpp-activated by a factor of 2-fold [19]. In relation to virulence we also note ppGpp-dependent activation of the sRNA, IsrH-2 (16-fold); this sRNA was found to be located within IsrH-1 and in accordance with our results, the former but not the latter sRNA was found to be expressed at stationary phase and to be highly induced within infected macrophages [73] (Table S8). Finally we note that the majority of stable RNA TSSs were ppGpp-repressed at LSP, apart from 7 tRNA TSSs which were ppGpp-dependently elevated greater than 4-fold. This was in contrast to ESP where the majority of tRNA TSSs (all but one) were ppGpp-dependently elevated greater than 4-fold. This is perhaps indicative of changes in the ppGpp-dependent differential processing or stability of tRNAs between ESP and LSP rather than any direct ppGpp-dependent regulation [19].

## Methods

### Bacterial Strains and RNA extraction

The virulent S. Typhimurium parent strain, SL1344 and the isogenic Δ*relA*Δ*spoT* strain were obtained as described in [19]. Bacterial cultures were grown overnight in Luria-Bertani broth (LB) at 37°C, 250 rpm, from -70°C glycerol stocks and used to inoculate into 50 ml of fresh LB in 250 ml conical flasks. The cultures were grown aerobically at 37°C with shaking at 250 rpm to an OD_600_ of 3.6 (defined in this study as later stationary phase; LSP), conditions previously shown to induce SPI2 gene expression [23], [24]. At this point, bacterial cultures were harvested and total RNA was extracted as described in [19]. The growth curves for SL1344 parent strain and the Δ*relA*Δ*spoT* strain, and time of harvesting are shown in Fig. S1.

### Library preparation and sequencing

In order to differentiate primary from processed transcripts, total RNA from biological replicates of each strain were divided into equal quantities and one half treated with Terminator 5′-phosphate-dependent exonuclease (TEX; Epicentre Biotechnologies) as previously described [19]. TEX specifically degrades RNAs with a 5′ phosphate end but does not degrade transcripts with a 5′ triphosphate end (primary transcripts) and thereby enriches for primary transcripts. The TEX treatment and cDNA library construction was performed by Vertis Biotechnology, AG, Germany (http://www.vertis-biotech.com) as described in [19]. For the Illumina-Solexa sequencing, four strand specific cDNA libraries were prepared: SL1344 parent non-enriched (SL1344-NE), SL1344 parent enriched (SL1344-EN), Δ*relA*Δ*spoT* non-enriched (DM-NE) and Δ*relA*Δ*spoT* enriched (DM-EN). Each library was tagged with a different barcode at the 5′ end to enable multiplexing during sequencing. Four of the Illumina-Solexa libraries were pooled and sequenced in a single lane using 50 single-read cycles on an Illumina Genome Analyzer II sequencing machine (GATC Biotech, Germany), yielding a total of ∼2×10^7^ reads.

### Analysis of sequences and statistics

The mapping statistics for the Illumina-Solexa cDNA libraries are shown in Table S5. Following sequencing, custom PERL scripts were used to separate the cDNA libraries based on their barcodes and to remove 5′ linker regions. Illumina-Solexa generated reads were mapped onto the *S.* Typhimurium SL1344 genome (Genbank ID: FQ312003.1) including the three native virulence plasmids (SLP1, SLP2 and SLP3) using the *segemehl* program [76]. Mapped reads were converted to a graph file and visualized on an Integrated Genome Browser (IGB) [77]. For comparison of expression levels between strains, mapped read numbers were normalised to their respective gDNA library sizes.

## Supporting Information

Figure S1 Growth curves for *S*. Typhimurium SL1344 parental and Δ*relA*Δ*spoT* strains.
(DOCX)

Figure S2 Single promoter rearrangements at LSP compared to ESP. Red arrows indicate LSP-specific TSSs.
(DOCX)

Figure S3 MEME analysis of 282 candidate LSP ncRNAs showing conserved -10 region (TATAAT).
(DOCX)

Figure S4 ppGpp-dependent location of *gltA* transcriptional start sites.
(DOCX)

Figure S5 Promoter architecture and ppGpp-dependency of SPI1.
(DOCX)

Figure S6 Heat map showing clustered expression levels of genes encoded within SPI1 and SPI2 from early to late stationary phase in *S*. Typhimurium SL1344 parent and Δ*relA*Δ*spoT* strains from microarray transcriptomic data.
(DOCX)

Table S1 Master table of TSSs and ppGpp-dependent expression levels for *S*. Typhimurium SL1344 ORFs at LSP.
(XLSX)

Table S2 TSSs with absent or altered positions at LSP compared to ESP.
(XLSX)

Table S3 TSSs and ppGpp-dependent expression of candidate LSP ncRNAs.
(XLSX)

Table S4 Expression levels and ppGpp-dependent expression rRNAs and tRNAs at LSP and ESP.
(XLSX)

Table S5 Mapping statistics for SL1344 parental and Δ*relA*Δ*spoT* libraries.
(XLSX)

Table S6 Location of SsrB binding sites relative to transcription start sites at SsrB dependent promoters.
(DOCX)

Table S7 ESP TSSs with no corresponding LSP TSS or alternative TSS.
(XLSX)

Table S8 TSSs and ppGpp-dependent expression of known and predicted sRNAs that have been validated by Northern blot analysis.
(XLSX)
