# Supplementary material for: The Primary Transcriptome of Salmonella enterica Serovar Typhimurium and Its Dependence on ppGpp during Late Stationary Phase
Source: PLoS One. 2014 Mar 24;9(3):e92690. doi: 10.1371/journal.pone.0092690 (PMC3963941; doi:10.1371/journal.pone.0092690)
Supplement: Figure S1 — Growth curves for S. Typhimurium SL1344 parental and ΔrelAΔspoT strains. (DOCX) [file pone.0092690.s001.docx]

**Figure S1**


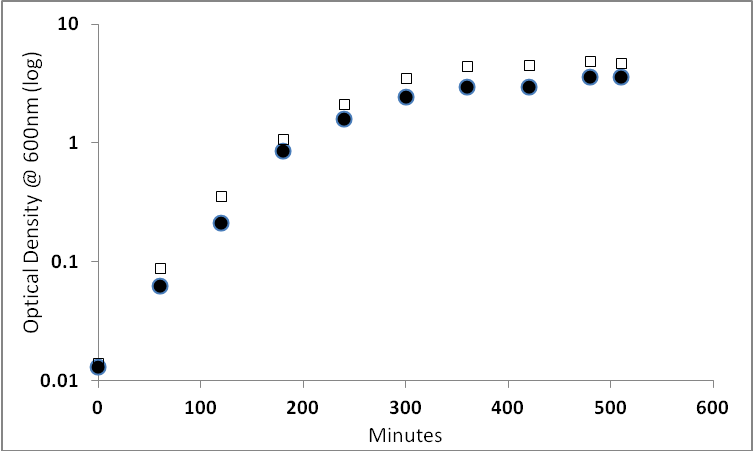


**Growth curves for *S*. Typhimurium SL1344 parental (closed circles) Δ*relA*Δ*spoT* strains (open squares).** Typical growth curves were performed on aerobically grown LB cultures at 37^o^C in a water bath shaking at 250 rpm. Under these conditions growth of parental and ∆*rel*A∆*spoT* strains were almost identical, however the ∆*rel*A∆*spoT* strain attained a slightly higher final density, in accordance with the findings of Pizarro-Cerdá and Tedin, 2004. *Molecular Microbiology,* **52**: 1827–1844. Samples for RNA extraction were removed at 240 minutes (black arrow) and processed according to Materials and Methods.
