# Supplementary material for: The Primary Transcriptome of Salmonella enterica Serovar Typhimurium and Its Dependence on ppGpp during Late Stationary Phase
Source: PLoS One. 2014 Mar 24;9(3):e92690. doi: 10.1371/journal.pone.0092690 (PMC3963941; doi:10.1371/journal.pone.0092690)
Supplement: Figure S2 — Single promoter rearrangements at LSP compared to ESP. Red arrows indicate LSP-specific TSSs. (DOCX) [file pone.0092690.s002.docx]

**Figure S2**

**Rearrangements of TSS positions at LSP compared to ESP.** Letters J, K, L, M, N, P and R indicate type of rearrangement. Red arrows indicate TSS positions identified at LSP but not at ESP. For further information and genes to which the reconfigurations apply see Table S2.
