# Supplementary figures and images for: The Primary Transcriptome of Salmonella enterica Serovar Typhimurium and Its Dependence on ppGpp during Late Stationary Phase
Source: PLoS One. 2014 Mar 24;9(3):e92690. doi: 10.1371/journal.pone.0092690 (PMC3963941; doi:10.1371/journal.pone.0092690)

**Figure S3**


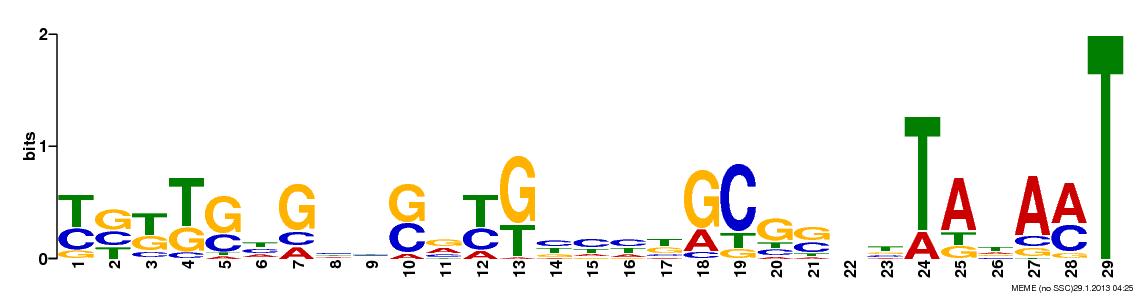


**MEME analysis of 282 candidate LSP ncRNAs showing conserved -10 region (TATAAT).**

Supplement: Figure S3 — MEME analysis of 282 candidate LSP ncRNAs showing conserved -10 region (TATAAT). (DOCX) [file pone.0092690.s003.docx]
