# Supplementary material for: The Primary Transcriptome of Salmonella enterica Serovar Typhimurium and Its Dependence on ppGpp during Late Stationary Phase
Source: PLoS One. 2014 Mar 24;9(3):e92690. doi: 10.1371/journal.pone.0092690 (PMC3963941; doi:10.1371/journal.pone.0092690)
Supplement: Figure S4 — ppGpp-dependent location of gltA transcriptional start sites. (DOCX) [file pone.0092690.s004.docx]

**Figure S4**

***gltA***

797002

(SL1344

parent)

797031

(ppGpp^0^)

**ppGpp-dependent location of *gltA* transcriptional start sites.** The parental and ppGpp^0^ TSSs are located at genomic coordinates 797002 and 797031 respectively. The TSS in the SL1344 parental strain matches to within 3 nt the S_1_ TSS defined for *gltA* by Wilde and Guest in *E. coli* (*J. Gen. Microbiol*., (1986), **132**: 3239).
