# Supplementary material for: The Primary Transcriptome of Salmonella enterica Serovar Typhimurium and Its Dependence on ppGpp during Late Stationary Phase
Source: PLoS One. 2014 Mar 24;9(3):e92690. doi: 10.1371/journal.pone.0092690 (PMC3963941; doi:10.1371/journal.pone.0092690)
Supplement: Figure S5 — Promoter architecture and ppGpp-dependency of SPI1. (DOCX) [file pone.0092690.s005.docx]

**Figure S5**

**Promoter architecture and ppGpp-dependency of *Salmonella* pathogenicity island 1 at LSP.** Enriched (+) and non-enriched (-) cDNAs of *S*. Typhimurium SL1344 parental (black) or *∆relA∆spoT* (red) strains mapped onto SPI1. The Y axis in each lane represents 0-50 mapped reads per genome position. Grey boxes represent individual SPI1 genes and operonic transcripts are indicated by black arrows and labelled according to the first gene of the operon as defined in [[1-6](#_ENREF_1)]. The vertical black and blue arrows indicate ppGpp-dependent TSSs at genomic coordinates 3039861 and 3047036 respectively; possible interpretations are described in the Discussion. The relevant genome co-ordinates span the centre of the figure.
