# Supplementary material for: The Primary Transcriptome of Salmonella enterica Serovar Typhimurium and Its Dependence on ppGpp during Late Stationary Phase
Source: PLoS One. 2014 Mar 24;9(3):e92690. doi: 10.1371/journal.pone.0092690 (PMC3963941; doi:10.1371/journal.pone.0092690)
Supplement: Figure S6 — Heat map showing clustered expression levels of genes encoded within SPI1 and SPI2 from early to late stationary phase in S. Typhimurium SL1344 parent and ΔrelAΔspoT strains from microarray transcriptomic data. (DOCX) [file pone.0092690.s006.docx]

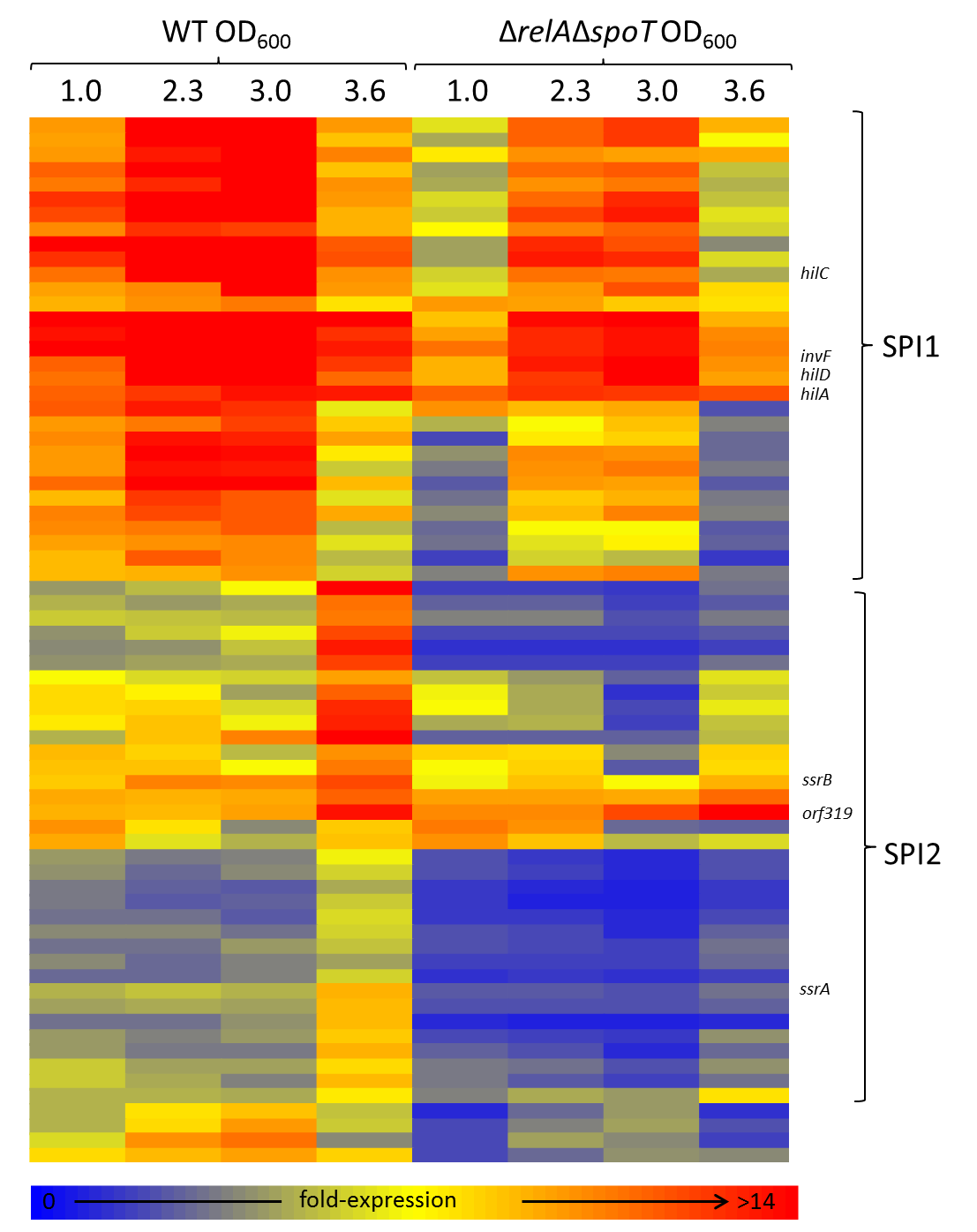


**Figure S6.** **Heat map showing clustered expression levels of genes encoded within *Salmonella* Pathogenicity Islands 1 and 2 from early to late stationary phase in *S*. Typhimurium SL1344 wild-type and ∆*relA*∆*spoT* strains.** The filtered data was clustered according to similarity of expression level using the standard correlation tool in GeneSpring 7.3^TM^ (Agilent). Each gene is colour-coded according to the level of expression (i.e. signal ratio of cDNA versus genomic DNA). Microarray analysis was essentially performed as described in Thompson *et al*., 2006 *J. Biol Chem* **281**:30112. Total RNA was extracted from *S.* Typhimurium SL1344 wild type and isogenic Δ*relA*Δ*spoT* strains as described above. The RNA was labelled and hybridised to IFR SALSA2 whole ORF microarrays (www.ifr.ac.uk/Safety/Microarrays/default.html#protocols), and data processed and analysed using GeneSpring™ (Agilent). The data was from 3 biological replicates, statistically filtered (P = 0.05) and a 2-fold cut off applied. Highly expressed genes are shown in red and weakly expressed genes are dark blue. The major island-encoded regulators of SPI1 and SPI2 are annotated (*hilA*, *hilC*, *hilD*, *invF*, *ssrA*, *ssrB*). The figure was compiled from microarray data deposited at NCBI's Gene Expression Omnibus (accession number GSE48478).
