# Supplementary material for: The Primary Transcriptome of Salmonella enterica Serovar Typhimurium and Its Dependence on ppGpp during Late Stationary Phase
Source: PLoS One. 2014 Mar 24;9(3):e92690. doi: 10.1371/journal.pone.0092690 (PMC3963941; doi:10.1371/journal.pone.0092690)
Supplement: Table S5 — Mapping statistics for SL1344 parental and ΔrelAΔspoT libraries. (XLSX) [file pone.0092690.s011.xlsx]

**Table S5. Mapping statistics for *S*. Typhimurium SL1344 and SL1344 ∆*relA*∆*spoT* libraries.**

| **LSP Sample** | **Total Reads** | **Mapped**  **reads** | **% mapped reads** | **Unmapped**  **reads** | **% unmapped reads** |
| --- | --- | --- | --- | --- | --- |
| SL1344_non-enriched | 6479669 | 3839617 | 59.26 | 2640052 | 40.74 |
| SL1344_enriched | 5867926 | 3624307 | 61.76 | 2243619 | 38.24 |
| ppGpp^0^_non-enriched | 3985265 | 3088392 | 77.50 | 896873 | 22.50 |
| ppGpp^0^_enriched | 3551597 | 2916865 | 82.13 | 634732 | 17.87 |

SL1344 – parental strain, ppGpp^0^ – SL1344 ∆*relA*∆*spoT*. See Methods for definitions of non-enriched and enriched libraries
