# Supplementary material for: The Primary Transcriptome of Salmonella enterica Serovar Typhimurium and Its Dependence on ppGpp during Late Stationary Phase
Source: PLoS One. 2014 Mar 24;9(3):e92690. doi: 10.1371/journal.pone.0092690 (PMC3963941; doi:10.1371/journal.pone.0092690)
Supplement: Table S6 — Location of SsrB binding sites relative to transcription start sites at SsrB dependent promoters. (DOCX) [file pone.0092690.s012.docx]

**Table S6. Location of SsrB binding sites relative to transcription start sites at SsrB dependent promoters.**

| **Gene name** | **Promoter regions showing predicted SsrB binding motifs.** |
| --- | --- |
| *ssaB* | AGTCGAAATAAATGTGAATCAGGCTTTTTACGGATGTGGTTGTGAGCGAATTTGATAGAAACTCCCATTTA -35 |
| *ssaG* | GCGCCGGAGTAATCGTTTTCAGGTATATACCGGATGTTCATTGCTTTCTAAATTTTGCTATGTTGCCAGTA -35 |
| *ssaM* | TCGTGAAGTAAAGATAAATCAGGTTTTATTCTGATACCTGGCTTTCAATATTTAGGTAAATTGGCTTTC -33 |
| *ssaR* | TTGGTATCTTTGCAAAAATACAGCGTAAACCTGATAAGAAAAATAATATGCGAACAATATAATAGCGTTC -34 |
| *sopD2* | GTCGCATTCAACGCGCAATCAGGTGTAAATCTGATGTCATTTCTAAACCCAGGCTGATTCAATCTCTTAAA -35 |
| *sifA* | CGTTTATACGCGAAGCTCTCAGGTTTTATACTGATTGCCAGTCTCTTTTAAAAATTATATTACATCCGATG -35 |
| *sifB* | TTCAGGACAAAGCAAAAATCAGGTGTTTCACCGATAGGCAAACCGATGGGCAACATGGGATAATATTTCGA -35 |
| *sseK2* | TCTTCCAATTTATTAAACTAAGGTTAAAACCCGATATCTTATCAATCTCAAATCATGGTATGTTATATTAA -35 |
| *sseL* | ATGTGAATAATAAGAAAATCAGGTCTATGCCTGATTTAATATATCCCCCGCTAATAATAATATTTTTACA -34 |
| *steC* | TATGAAAAATTTTGGAAATCAGGTGATTACAGTAGTTACAAATCAGGTTTTTATATGTTTGCATGTGTA -33 |
| *sseA***’** | ACAGGATCCGCAGCAATATCAGCAAAACACCTTATTGCTTGAAGCGATCGAGCAGGCCGAAAATATCATCA -35 |
| *sspH2* | TGTCAGGACGACCATGAATCCGCCGATAACCTTATTGTGAAATTAAGACCAGGAAGAGATGATGTCTGCC -34 |
| *sseJ* | AAGAAGCGTAATTCCATATACACCATTTACCTGATTACTTTTCTTGCTAATATTTGCTAATTAATTATTTG -35 |
| *pipB2* | TATGTAACCAGACGTAAAGGGGGTATTCACCTTATCTCTAAATGCAAATCTATATGATAAATTTTATCATG -35 |
| *srcA* | ATTTCAAACACCATAATATCAGATAAAAACCTGATATTTATTACCAAACATACTTTTTAATAGCATAAAATCGAAA  TGTTCTTTAGACTCTTTAGCA -62 |
| *ssrB* | GGCTGGAGAAAAAATCGATCAGTTAATTCACACATTAAAAGGCTGTTTAGGTCAAATAGGGCAGACTGAATTGGTATGCTATGTCATAGACATTGAGAATCGCGTAAAAATGGGGAAAATCATCGCGCTGGAGGAACTAACCGACTTACGCCAGAAAATACGTATGATCTTCAAAAACTACACCATTACTTAATATTATCTTAATTTTCGCGAGGGCAGCAAAATG -38 |
| *sseI* | TCCCCAGCGTTAATTCTATGCGCCAGTCCTTAATGGCATTATCTGAATCGTTAAGTAATTTCTGCCGGGTGACTTC  TGCTGCCAAAATTGAACGCCGGGCGTTGATACCGGATGATCTGGTCATCGTGGAAAGCGACCCTGAAAA**A**ATCGAC  ACTTAGCTGTAAAATGACAGTCCCGCCATCCGGTCATCATAACGGATTTTTCTTCTGCACCTTCTGAAGCCCGCCA  TGGCAGGACGACCATGAATCCGCCGATAACCTTATTGTGAAATTAAGACCAGGAAGAGATGATGTCTGTCGGACAG  ATACTATATGTAAATTTATAAAGGTTTTTTGTTATG -34 |
| *pipB* | TGTAAGTATTCCTGCTCATCAGGTTTTTACGCCATCTACGCGCATTTATTCTGGTATAAGTTGAAATACTGCAAAAAATATTGGTGCTTATTATTTTTTCTTTAAGTAAATTTTCGCTCAACAAACTTAATTGTTTATTCAATGATGATGAAGCGTAAGCTATGCTGGAAATGAAGGAAGTCAATAGCAAGGATAATCTTATTATTCACGGGTGATATTACTTCTGCTTCACCGTTATGGCAGATATCATCGCCTCTTGTCAGATGCCAGACACCTACTCATACTCAACCAAAGCTCTAAATACAAAAATCACCTTATATCTTTTTTTATTATTCCTTGTATAAATGTGACT -137,-316 |
| *steB* | ATCAATAGCTTATAGATTTCCGGCATAGACCATCAAGAATCAGTTCACATTCAGCATATTCAGCTCATGGGAACTTTTTCAGGCCACAGACAATAAACACCAAAGCTTTCTTTCTAAGCTGCAATGTTCTGCCCGACTGGGGCACAGAGGTAATGCCGGTAAATCCAGTCCCATTACCCAGAAGTAAATATTTGCTTAATAGTTTTTATTTACTCACCAGGTGGTAATGAATAAAGGTTATGATATGCAGA -215 |
| *slrP* | ATATCCTTATCTGTTACTTTAGGTTACGTTCAGATCAGGTAGGGAAAATATG |
| *sseA* | GTTCAAAATGGCAAGTAAAATAGCCCTAATGGGATAGGCTCTTAGTTAGCACGTTAATTATCTATCGTGTATATGGAGGGGAATGATGATAAAGAAAAAGGCTGCGTTTAGTGAATATCGTGATTTAGAGCAAAGTTACATG |
| *ssrA* | CTATACTCTTCTTGTACAATATCAGGATGCTGTCTACATATACCTTGTCACAGGCGATTCTATCATTCGGATTTTCCGATAAATTCACAATTACATTTTCAGCATTGACATAAAAACTTACAATTTGAAAAATTATTTATTAAATAAACTGTTACGATGTTTTTACATCGCCATCTTATTAAAAAGTAATTGTAGTCATCGACTGGGTTATATATGAAGAAATTTATCTTCCTAATGATAACACCATCGATTAATCTTCTGATGAAACTATATGTACTGCGATAGTGATCAAGTGCCAAAGATTTTGCAACAGGCAACTGGAGGGAAGCATTATG +80, +234 |

The above Table shows maps of the SsrB regulated promoters containing SsrB binding sites (identified in [5]) for which TSS information is also available. The SsrB binding sites are shown in red blocks, TSSs are shown in yellow blocks. Following alignment of promoter regions we were able to visually identify two ‘new’ potential SsrB consensus binding sites (as defined in [5]), approximately 35 nucleotides upstream of the *steC* and *ssrB* TSSs, and these are shown in red text. Translational start codons are underlined and DNase I footprinted SsrB binding sites that do not overlap the sites identified in [5] are shown in blue text. The number of nucleotides between the consensus SsrB binding motif and the TSS is given at the end of each sequence. TSSs were previously reported for *ssaB, ssaG*, *ssaM, sseA* [6], *sifA*, *sifB, sseJ* [6], *ssrA*, *ssrB* [1], *sseI* [2], *sseK3*, *sseL*, *steB*, *slrP,sseA* [4]. The remainder of TSSs were identified from this work; the dRNA-seq identified TSSs for *sopD2*, *sspH2* and *sseI* are not entirely certain but are included for comparison since they are all located 34-35 nucleotides downstream of SsrB binding sites.

**Notes:**

- For the *steC* promoter the SsrB binding site identified in [5] is shown as well as second partial SsrB consensus binding site we identified immediately upstream of the -35 element (red text).
- For the *sseL* promoter a TSS has previously been reported [3], and was found to be located 27 nt downstream of the TSS identified in this study.
- The *sseA****’*** promoter region shows the TSS we previously identified within the 3’ end of the SsaE CDS [4], whereas the *sseA* promoter sequence shows the 2 TSSs identified in the *ssaE*-*sseA* intergenic region by Walthers *et al.,* (2007) [6] but not observed in our LSP dRNA-seq data.
- *srcA* is co-transcribed from a promoter upstream of SL1344_2115.
- For *sseI a* TSS was previously identified (shown as a bold ‘A’ in the above Table), and an SsrB binding site was identified by DNase I footprinting [2]; the LSP dRNA-seq data showed no evidence of a TSS at this position although a potential TSS was observed 34 nt downstream of the SsrB binding site identified in [5].
- The TSS identified in [1] for *ssrB* was not observed in the LSP dRNA-seq data but was included here since we identified a potential consensus SsrB binding site 38 nucleotides upstream of it. The SsrB binding site determined by DNase I footprinting in [1] is shown in blue font.

**References**

1. **Feng, X., R. Oropeza, and L. J. Kenney.** 2003. Dual regulation by phospho-OmpR of ssrA/B gene expression in *Salmonella* pathogenicity island 2. Molecular microbiology **48:**1131-1143.

2. **Feng, X., D. Walthers, R. Oropeza, and L. J. Kenney.** 2004. The response regulator SsrB activates transcription and binds to a region overlapping OmpR binding sites at *Salmonella* pathogenicity island 2. Molecular microbiology **54:**823-835.

3. **Gal-Mor, O., D. Elhadad, W. Deng, G. Rahav, and B. B. Finlay.** 2011. The Salmonella enterica PhoP directly activates the horizontally acquired SPI-2 gene sseL and is functionally different from a *S. bongori* ortholog. PloS one **6:**e20024.

4. **Ramachandran, V. K., N. Shearer, J. J. Jacob, C. M. Sharma, and A. Thompson.** 2012. The architecture and ppGpp-dependent expression of the primary transcriptome of *Salmonella* Typhimurium during invasion gene expression. BMC genomics **13:**25.

5. **Tomljenovic-Berube, A. M., D. T. Mulder, M. D. Whiteside, F. S. Brinkman, and B. K. Coombes.** 2010. Identification of the regulatory logic controlling *Salmonella* pathoadaptation by the SsrA-SsrB two-component system. PLoS genetics **6:**e1000875.

6. **Walthers, D., R. K. Carroll, W. W. Navarre, S. J. Libby, F. C. Fang, and L. J. Kenney.** 2007. The response regulator SsrB activates expression of diverse *Salmonella* pathogenicity island 2 promoters and counters silencing by the nucleoid-associated protein H-NS. Molecular microbiology **65:**477-493.
